# A ‘Final Destination injury’: Penetrating trauma of the neck and a pneumomediastinum by a metal part shot from a lawnmower

**DOI:** 10.1016/j.tcr.2020.100379

**Published:** 2020-12-11

**Authors:** Marcel L.J. Quax, Daniel Eefting, Jeroen C. Jansen, Joris J. Blok

**Affiliations:** a Departments of Vascular Surgery, Leiden University Medical Center, Leiden, the Netherlands; b Departments of Otorhinolaryngology, Leiden University Medical Center, Leiden, the Netherlands

**Keywords:** Penetrating injury, Neck injury, Neck trauma

## Abstract

**Introduction:**

Outside of war regions, penetrating neck injury is rare. Penetrating neck injury due to a lawnmower has never been described, despite the annual 74.000 injuries caused by lawnmowers in the United States. In this report, the case of a 65-year old women, admitted after a penetrating neck injury due to a metal piece shot from a lawnmower, is described.

**Report:**

A 65-year old women, with no relevant medical history, presented at the Emergency Department after she was hit in the neck by an iron projectile shot from a professional lawnmower. On site, the projectile, a metal part from the lawnmower blade, was removed by her husband. CT scan showed a pneumomediastinum, without signs of vascular injury. Surgical exploration was immediately performed in the operation room (OR). No vascular or esophageal injuries werefound, only lacerated neck muscles. Perioperatively, the ENT surgeon performed an endoscopy, which showed a small injury of the hypopharynx. Postoperatively, the patient was prophylactically treated with antibiotics for 7 days. Patient was discharged in good clinical condition after 7 days, without complications.

**Conclusion:**

In this report we present a case with a sharp traumatic injury of the neck, caused by a metal projectile shot from a lawnmower. The laceration of the pharynx was explored in the OR by the ENT- and vascular surgeon. The pneumomediastinum was treated with prophylactic antibiotics. Currently the patient is doing fine without any complications of the injury. Traumatic injury of the neck requires direct direct surgical exploration, however, when patients present hemodynamically stable, a neck CTA will add to the diagnosis (e.g. a pneumomediastium).

## Informed consent

Informed consent was obtained from the patient involved. The authors of this manuscript have no financial or personal relationships with organizations that inappropriately influence their work.

## Introduction

Lawnmowers are dangerous, as is reflected by the annual 74.000 hospital visits that were registered in the United States in 2004 as a result of a lawnmower injury. Most of the injuries included foot fractures, or soft tissue injury of the extremities as a result of getting hit by a (part of a) lawnmower. [1]

Penetrating injury of the neck caused by a lawnmower has never been described before. Shrapnel war injuries, stab wounds or perforation following high energetic trauma are most described in literature as a mechanism of penetrating neck injury. In war injuries mortality due to these types of injury ranged from 18% to 23%. [[2], [3], [4]]

In this report, we describe a rare case of penetrating neck injury caused by a metal part shot from a lawnmower.

## Case report

**Fig. 1:**
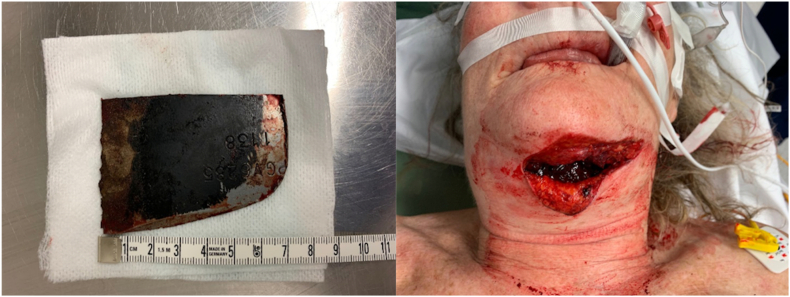
The metal part that was shot by a lawnmower (left) and the laceration at primary survey in the emergency department (right).

A 65-years old women, without relevant medical history, presented at the Emergency Department of the Leiden University Medical Center. While riding her bicycle, she was struck by a metal piece that was shot from a lawnmower across the street. After removal of the metal part from the lawnmower blade (Fig. 1) on site by her husband, she remained hemodynamically stable and was brought to the hospital by ambulance.

**Fig. 2:**
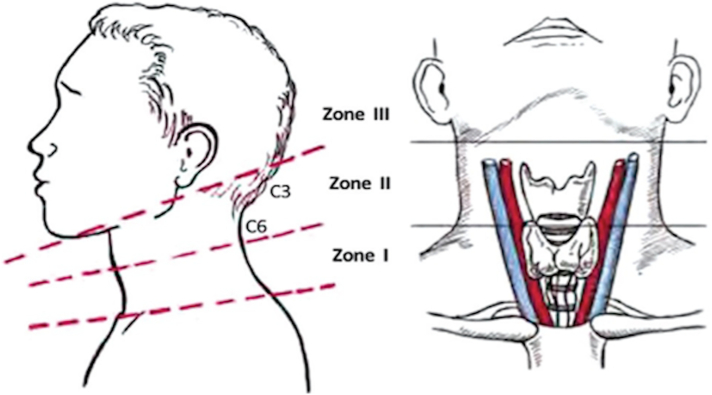
Neck regions (figure from; Ucak M Shrapnel Injuries on Regions of Head and Neck in Syrian War. J Craniofac Surg. 2020 Mar 23.).

At primary survey, she had some blood at the tongue base, without life threatening airway problems. Cranioventrally in zone 2 of the neck (Fig. 2), the patient had a laceration of approximately 10x10cm, without obvious arterial bleeding. The oxygen saturation was 100% without additional oxygen, her breathing frequency was 12 per minute and there were no chest injuries. With a he heart rate of76 bpm and a stable blood pressure of158/100 mmHg there was no suspicion of circulation problems. No additional neurological or extremity injuries were found.

Laboratory results showed no abnormalities; hemoglobin was 8.5 mmol/L (normal range: 7.5-10 mmol/L). A computed axial tomography angiography (CTA) of the neck and chest showed no signs of arterial or venous damage. However, there was extensive soft tissue damage, with a suspicion of an injury of the posterior wall of the pharynx with air subcutaneously extending into a pneumomediastinum. Patient went to the operation theatre for exploration by the vascular surgeon and ear-nose-throat surgeon.

**Fig. 3:**
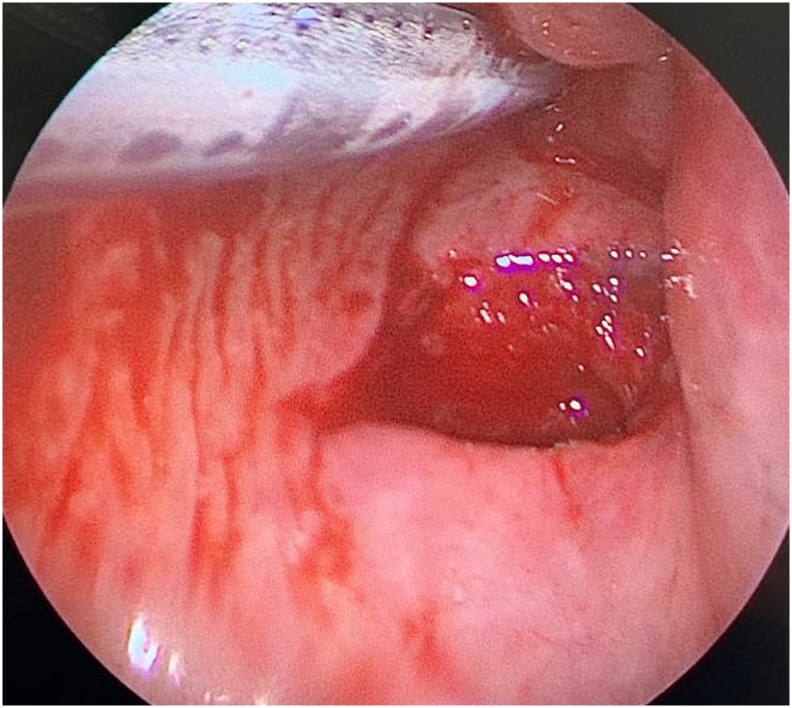
Endoscopy image with small injury of the left vallecula.

After intubation, endoscopic inspection of the pharynx showed a small injury of the left vallecula. A second laceration of the posterior wall marked the spot where the penetrating metal was stopped by the cervical spine (Fig. 3). Both injuries were left to secondary healing.

During surgical exploration several small venous bleedings were found and ligated. The platysma muscle, omohyoid muscle, thyrohyoid and sternohyoid muscles were sliced by the injury and approximated with absorbable sutures. There was no post-traumatic injury of the trachea or hypoglossal- and vagal nerves, nor were the carotid artery or jugular vein dissected. Hemostasis was reached with diathermia and closure of the wound in anatomical layers. At the end of the procedure, a nasogastric tube was inserted to start feeding).

**Fig. 4:**
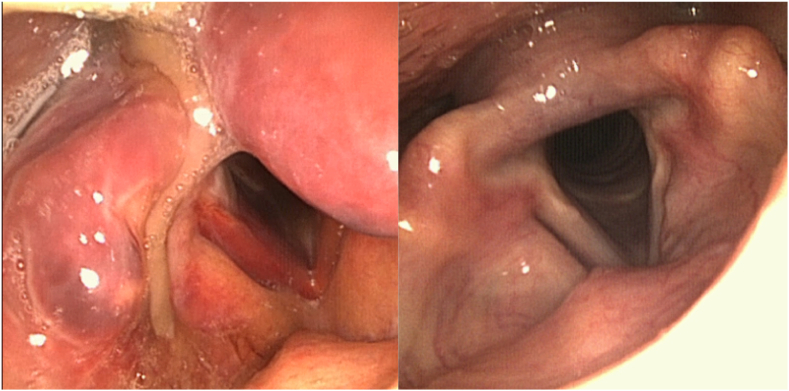
Perioperative fiber endoscopy images; edema of the pharynx.

Postoperatively, the patient was treated prophylactically with intravenous amoxicillin/clavulanic acid for 7 days. Fiber endoscopy on day 3 showed laryngeal edema and hematoma (Fig. 4). On postoperative day 3, the patient started with liquid oral feeding. On day 4, the nasogastric tube was removed and the patient started eating solid food. CT showed no increase of the pneumomediastinum at postoperative day 5. On day 7, the antibiotic treatment was ceased and the patient was discharged from the Surgical Ward.

At follow-up after one week, the patient was doing well, apart from some mild complaints when swallowing solid food. Follow-up at four months after the event complete resolution of the laryngeal and pharyngeal sequalae was observed and the patient is doing fine.

## Discussion

In this report we present a case with a sharp traumatic injury of the neck, caused by a metal projectile shot from a lawnmower. The trauma mechanism described, is quite rare when evaluating the literature of penetrating neck injuries. Traumatic lawnmowers injuries are more often found in children and less described in adults. Most of these injuries are caused by direct contact with a spinning blade and less frequently by projectiles propelled by the blade, let alone a part from the blade itself. In the scarce available literature there are some reports describing penetrating injury due to lawnmower projectiles, leading to abdominal aortic injury [5] or a mediastinal perforation. [6] Of these reports describes the death of one patient who was found in the grass, whereas other patients were discharged within 4 days postoperatively. [5.6].

The mechanism of presented injury is most comparable to shrapnel injury of the neck - a high velocity sharp iron piece is shot as a projectile into the neck – with a potentially high risk on injury of the vascular and nervous structures or, aerodigestive tract In general, suturing of shrapnel injuries of the pharyngeal wall is not necessary, as these injuries need surgical flap reconstruction in 49% of the cases. [2] The are no articles published on penetrating trauma after a projectile shot by a lawnmower, which means this trauma mechanism combined with the injury is uncommon or at least rare.

In 2007 Bell et al. wrote an overview of traumatic neck injuries and their treatments. Their analysis of penetrating neck injuries showed that injury at the external jugular vein and facial- or cervical spine fractures occurred most frequently, followed by the external carotid artery and injury of the hypopharynx. They described a decreased urge of exploration, when no injuries are suspected in CTA [7].

Prichayudh et al. also described the management of penetrating neck injury. Since the introduction of the “no-zone” approach of penetrating neck injury, the amount of negative surgical explorations decreased. The “no-zone” approach means that treatment is not based on anatomical location of the injury, but on the symptoms and diagnostics. In patients with “hard signs”, including signs of vascular injury (i.e., active bleeding and expanding hematoma) and aerodigestive injury (i.e., massive subcutaneous emphysema and air bubbling through the neck wound), were taken directly to the operating room for immediate neck exploration. In stable patients of asymptomatic patients, additional diagnostic investigation has to be performed to rule out internal organ injuries. [8]

In the current case the patient was hemodynamically stable, which allowed us to first perform a CTA. This revealed a pneumomediastinum and the indication for a laryngoscopy by the ENT surgeon.

A pneumomediastinum may result from any of these four mechanisms: direct air leak from rupture of aerodigestive system in the mediastinum; tears in the pulmonary parenchyma resulting in leakage of air into the pulmonary interstitium; air can further dissect along the fascial sheaths and the adventitia of blood vessels and bronchi into the mediastinum; or perforation of a hollow abdominal viscus with subsequent dissection of air into the mediastinum via the diaphragmatic hiatus. [9] In our case air dissected out of the mucosal injury of the pharynx and probably moved into the mediastinum. In a spontaneous pneumomediastinum, conservative treatment is often followed. In secondary pneumomediastinum due to trauma, antibiotics are given to prevent for mediastinitis. Kornmann et al. wrote a case report with penetrating neck injury (neck zone 1) with a pneumomediastinum due to a penetration of the mediastinum. Patient was treated with antibiotics 7 days postoperatively. [10] In our case, there was no direct penetration of the mediastinum, which differs in the direct risks on infection after the injury.

## Conclusion

In conclusion, we presented a case where a sharp injury of the neck, caused by a metal projectile (part of the lawnmower blade) shot from a lawnmower, only causing a laceration of the pharynx and a pneumomediastinum. After CTA, the wound was explored in the OR. The ENT-surgeon performed a laryngoscopy, which revealed two small lacerations, for which a consercvative treatment was followed. The pneumomediastinum was treated with prophylactic antibiotics for 7 days to prevent a mediastinitis. In future cases with traumatic, penetrating neck injury we would advise to perform a CTA in hemodynamically stable of asymptomatic patients, with surgical exploration of the wound when injuries are suspected. Direct surgical exploration is indicated when hard signs of internal injury are present at the ED. The treatment is multidisciplinary. Treatment of mediastinitis is often conservative with the use of antibiotics. Currently the patient is doing fine without any complications of the injury.
